# Type 2 diabetes remission: 2 year within-trial and lifetime-horizon cost-effectiveness of the Diabetes Remission Clinical Trial (DiRECT)/Counterweight-Plus weight management programme

**DOI:** 10.1007/s00125-020-05224-2

**Published:** 2020-08-10

**Authors:** Yiqiao Xin, Andrew Davies, Andrew Briggs, Louise McCombie, C. Martina Messow, Eleanor Grieve, Wilma S. Leslie, Roy Taylor, Michael E. J. Lean

**Affiliations:** 1 Health Economics and Health Technology Assessment, Institute of Health and Wellbeing, University of Glasgow, Glasgow, UK; 2 Department of Health Services Research and Policy, London School of Hygiene and Tropical Medicine, London, UK; 3 Human Nutrition, School of Medicine, Dentistry and Nursing, College of Medical, Veterinary and Life Sciences, University of Glasgow, Level 2, New Lister Building, Glasgow Royal Infirmary, 8-16 Alexandra Parade, Glasgow, G31 2ER UK; 4 Robertson Centre for Biostatistics, University of Glasgow, Glasgow, UK; 5 Newcastle Magnetic Resonance Centre, Translational and Clinical Research Institute, Campus for Ageing and Vitality, Newcastle University, Newcastle upon Tyne, UK

**Keywords:** Caloric restriction, Cost benefit, Cost-effectiveness, Type 2 diabetes, Weight loss

## Abstract

**Aims/hypothesis:**

Approximately 10% of total healthcare budgets worldwide are spent on treating diabetes and its complications, and budgets are increasing globally because of ageing populations and more expensive second-line medications. The aims of the study were to estimate the within-trial and lifetime cost-effectiveness of the weight management programme, which achieved 46% remissions of type 2 diabetes at year 1 and 36% at year 2 in the Diabetes Remission Clinical Trial (DiRECT).

**Methods:**

Within-trial analysis assessed costs of the Counterweight-Plus intervention in DiRECT (including training, programme materials, practitioner appointments and low-energy diet), along with glucose-lowering and antihypertensive medications, and all routine healthcare contacts. Lifetime cost per quality-adjusted life-year (QALY) was estimated according to projected durations of remissions, assuming continued relapse rates as seen in year 2 of DiRECT and consequent life expectancy, quality of life and healthcare costs.

**Results:**

Mean total 2 year healthcare costs for the intervention and control groups were £3036 and £2420, respectively: an incremental cost of £616 (95% CI –£45, £1269). Intervention costs (£1411; 95% CI £1308, £1511) were partially offset by lower other healthcare costs (£796; 95% CI £150, £1465), including reduced oral glucose-lowering medications by £231 (95% CI £148, £314). Net remission at 2 years was 32.3% (95% CI 23.5%, 40.3%), and cost per remission achieved was £1907 (lower 95% CI: intervention dominates; upper 95% CI: £4212). Over a lifetime horizon, the intervention was modelled to achieve a mean 0.06 (95% CI 0.04, 0.09) QALY gain for the DiRECT population and mean total lifetime cost savings per participant of £1337 (95% CI £674, £2081), with the intervention becoming cost-saving within 6 years.

**Conclusions/interpretation:**

Incorporating the lifetime healthcare cost savings due to periods of remission from diabetes and its complications, the DiRECT intervention is predicted to be both more effective (QALY gain) and cost-saving in adults with type 2 diabetes compared with standard care. This conclusion appears robust to various less favourable model scenarios, providing strong evidence that resources could be shifted cost-effectively to support achieving remissions with the DiRECT intervention.

**Trial registration:**

ISRCTN03267836

**Figure:**
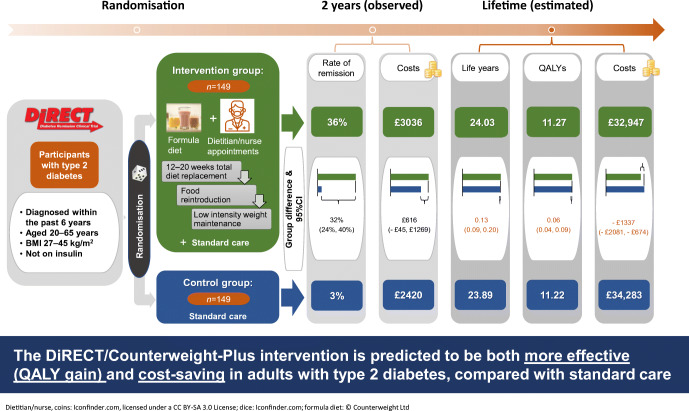
Graphical abstract

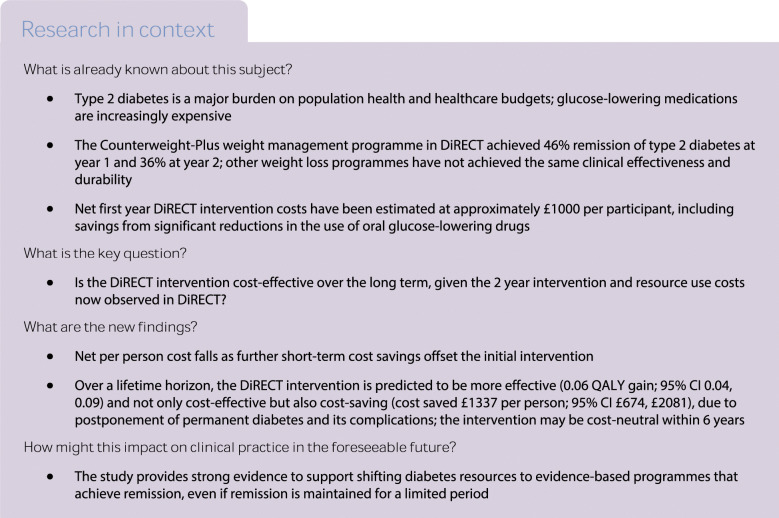



## Introduction

Type 2 diabetes, until recently considered a permanent and inevitably progressive chronic disease, impacts rates of mortality and morbidity as well as quality of life (QoL). Affecting between 5% and 35% of post-industrial adult populations [1], it presents a major and increasing economic burden, currently accounting for 10% of total healthcare expenditure in the UK [2] and 12.5% in the USA [3]. Management has usually focused on pharmacotherapy, with increasing emphasis in guidelines on earlier prescription of more modern and expensive glucose-lowering, lipid-lowering and antihypertensive drugs, to control blood glucose and reduce the associated complications and elevated cardiovascular risks. Despite these treatments, younger people, in particular, commonly face irreversible declines in health from type 2 diabetes, characterised by chronic pain and multiple disabilities, and life expectancy is reduced substantially [4]. However, remission of type 2 diabetes is now known to be possible [5–7]. In the Diabetes Remission Clinical Trial (DiRECT), an integrated diet programme delivered entirely within primary care produced remissions of type 2 diabetes (non-diabetic HbA_1c_ on no glucose-lowering medication) in 46% of participants at 1 year and 36% at 2 years [8, 9].

The DiRECT study, reported in detail elsewhere [8, 10], recruited 298 adults with a diagnosis of diabetes within the past 6 years, BMI 27–45 kg/m^2^ and HbA_1c_ >48 mmol/mol (6.5%), or >42 mmol/mol (6.0%) if receiving glucose-lowering medication. Half the participants were in practices randomised to receive the Counterweight-Plus weight management programme, and all received usual care under current clinical guidelines. The Counterweight-Plus programme is initiated as ‘total diet replacement’ with a low-energy formula diet (soups and shakes) providing 3452–3569 kJ (825–853 kcal) per day for 12–20 weeks (Counterweight, UK). This is followed by structured food reintroduction for 2–8 weeks and a subsequent longer-term programme of weight loss maintenance. For relapse management, 2–4 week ‘rescue packages’ of the formula diet are provided if >2 kg weight is regained or if diabetes returns. All oral glucose-lowering and antihypertensive medications are suspended on safety grounds at the start of the programme but are reintroduced according to blood glucose and blood pressure measurements taken at each primary care appointment, following clinical guidelines.

Remission of type 2 diabetes has the potential to lead to substantial long-term health gains and cost savings. Decision analytic models are commonly used to extrapolate long-term costs and outcomes from clinical trials, supplementing trial data with other sources of evidence on longer-term disease progression such as diabetes relapse, health-related QoL, mortality and costs [11]. Here we report a lifetime cost-effectiveness analysis of the Counterweight-Plus intervention, based on resource use measured in DiRECT over 2 years, and projected longer-term cost and quality-adjusted life-years (QALYs), assuming a limited duration of diabetes remission.

## Methods

A within-trial cost analysis was conducted using the 2 year data from DiRECT, including both the intervention costs and routine healthcare resource use measured during the time course of the study, for all participants (including those who did not complete the trial or who were unsuccessful in achieving remission). Lifetime cost-effectiveness was then estimated by predicting time to relapse (i.e. re-emergence of diabetes, assumed to be permanent thereafter) among those who were in remission at 2 years, applying the mean management costs for type 2 diabetes incurred within the UK National Health Service (NHS), under clinical guidelines which tend to favour older, less expensive medications. An NHS perspective for costing was adopted for both within-trial and lifetime analyses. Costs are presented in 2018 UK prices (£).

### Ethics approval

Ethics approval was granted in January 2014 by the West of Scotland Research Ethics Committee 3, with approvals by the NHS health board areas in Scotland and by clinical commissioning groups in Tyneside, UK.

### Two year within-trial economic analysis

DiRECT intervention costs over the 2 year trial period included costs for initial training of practitioners (dietitians or nurse practitioners, who each received a total of 16 h face-to-face training from Counterweight-Plus instructors), sachets of low-energy formula diet, practitioner monitoring appointments and the tailored Counterweight-Plus workbooks issued to each participant (Table 1). Costs of the sessions included practitioners’ attendance time and standard Counterweight-Plus materials. The number of sachets issued to each participant and the number and duration of practitioner appointments were collected prospectively throughout the study. Full costs for participant Counterweight-Plus workbooks were applied irrespective of participants’ persistence with the programme, the details of which are described elsewhere [8, 10, 12].

**Table 1:** Intervention resource use components and cost (per participant) (*n* = 149) over the first 2 years of the DiRECT trial

Intervention cost components	Quantity	Unit cost^a^ (£)	Total cost (£)	95% CI^b^
Intervention set-up cost (overall)				
Counterweight-Plus specialist training, support and mentoring	33 practitioners	300/practitioner	9900	
Practice nurse and dietitian time	16 h/practitioner	42/h	22,176	
Total set-up cost			32,076	
Total set-up cost annualised over 5 years^c^			7104	
Total intervention set-up cost per participant			48	
Intervention running resource use and costs (per participant)^d^				
Sachets issued		20/14 sachets		
Year 1	495 (458, 532)		708	654, 760
Year 2	95 (76, 112)		130	104, 155
Overall	590 (539, 639)		838	766, 908
Practice nurse or dietitian visits^d^		42/h (25–35 min/appointment)		
Year 1	15.6 (14.6, 16.6)		362^e^	337, 384
Year 2	7.7 (6.7, 8.6)		144^e^	125, 164
Overall	23.0 (21.4, 25.1)		506	464, 545
Counterweight-Plus booklets (year 1 only)	1	20/participant	20	–
Total intervention running cost per participant			1364	1260, 1464
Total intervention cost per participant (*n* = 149^f^)				
Year 1			1137	1071, 1205
Year 2			274	234, 313
Overall			1411	1308, 1511

^a^Unit cost £42/h for practice nurse or dietitian visits [13]; unit cost for intervention materials obtained from the DiRECT trial team

^b^95% CI from 1000 bootstrap iterations

^c^Annualised total cost over 5 years: *K*/[(1 − 1/(1 + *r*)^*n*^)/*r*], where *K* = £32,076, *r* = 3.5%, *n* = 5

^d^Values in the ‘Quantity’ column are mean (95% CI)

^e^Costed based on recorded duration of contacts (in minutes); year 2 cost discounted

^f^Includes six randomised participants who did not initiate the intervention

Details of all primary and secondary care visits for each of the participants were obtained directly from the participating general practitioner (GP) practice records. The costs of these were calculated using the recorded duration of contact for each appointment. Medication use was costed based on dose, frequency and start and end dates of individual participants’ medication records in each participating GP practice. Hospitalisation costs were estimated by matching reason for admission and recorded length of stay in DiRECT to the appropriate NHS reference cost (excluding excess bed-days, where length of stay recorded in DiRECT exceeded the national average). Unit costs were obtained from published national sources (Personal Social Service Research Unit [13], NHS reference costs [14], or Information Services Division Scotland [15], and British National Formulary [16]) and have been reported previously [17].

Statistical analysis was conducted based on the intention-to-treat principle. Missing data were minimal, as all resource use data were obtained directly from participating GP practices. Five intervention (3%) and three control (2%) participants relocated with loss to follow-up during the study. Their medication use was assumed to continue as at their last available records, and other healthcare resource use was assumed to be zero after relocation. Twenty intervention (13.4%) and six control (4%) participants had no data available for the 2 year remission outcome. In line with the primary outcome analysis, these participants were assumed not to have achieved remission.

Mean costs were calculated for each group, with clustering-adjusted SEs for each cost item. Incremental cost per remission at 2 years was reported as the difference in the groups’ total 2 year costs, divided by the difference in diabetes remission rates. All analyses were undertaken in Stata/MP, version 14.2 (StataCorp LP, USA), with 95% CIs based on 1000 non-parametric bootstrap iterations.

### Long-term projection

Long-term outcomes were projected for each treatment arm in DiRECT. A three-state model (remission, diabetes, death) was constructed. Individuals enter the model with existing diabetes. After 1 year a proportion achieve remission but are subject to relapse in future years. The proportion remaining in remission over time was estimated based on the rate of relapse observed in year 2; however, all participants were assumed to relapse after a given number of years (a maximum period of 10 years of remission in the base case). Life expectancy was calculated for each potential year of remission by applying rates of mortality for people free of diabetes up to the year of relapse and with diabetes thereafter. Along with life expectancy, quality-adjusted life expectancy and healthcare costs were estimated conditional on each potential year of relapse.

Life expectancy was calculated based on rates of mortality in people free of diabetes (*N* = 2.75 million) and with diabetes (*N* = 272,597), based on a recent UK study [18] which reported mortality rates by sex and 5 year age bands during the period 2012–2014 in Scotland. Reported life expectancy for men aged 55–59 years was 23.0 and 26.0 years with diabetes and free of diabetes, respectively, and 24.4 and 28.7 for women.

QALYs were calculated by applying standard UK age-dependent health state utility population norms [19]. These were assigned directly to people in remission from diabetes. For people not in remission, including those who had relapsed, these age-dependent health state utilities were reduced using a constant multiplier of 0.925 to reflect a decrement due to diabetes. This was estimated based on the mean population score (0.828) and the regression coefficient for diabetes (−0.0621) in the US Medical Expenditure Panel Survey catalogue of UK EuroQol EQ-5D scores [20]. This estimate was employed rather than being based on data from DiRECT, for reasons discussed below.

The lifetime healthcare costs associated with diabetes were compiled first from the measured costs for the first 2 years of DiRECT, to which were added further costs for ongoing weight management for participants remaining in remission and long-term healthcare costs associated with diabetes. Long-term diabetes-related healthcare costs were assumed to increase linearly with duration of diabetes (i.e. time since relapse), over 15 years, from £1250 in the year of diagnosis to £3117 after 15 years, based on a UK cost of diabetes study [2, 21]. No further increase in long-term healthcare costs beyond 15 years was applied. Long-term healthcare costs due to diabetes were applied to the proportion of people projected to be in the diabetes state each year after year 2.

We compared the incremental cost and QALYs for the intervention and control arms over the 2 year follow-up of DiRECT and over a lifetime horizon. Sensitivity analysis was undertaken for the long-term analysis, including exploring the impact of alternative relapse rates and maximum assumed durations of diabetes remission. Probabilistic sensitivity analysis was performed using 1000 bootstrap iterations of the DiRECT data (remission and 2 year costs), and Monte Carlo simulations for other variables (e.g. long-term mortality rates). Model input data are summarised in Table 2; all costs and outcomes beyond year 1 were discounted at the standard UK annual rate of 3.5%.

**Table 2:** Variable values for the long-term economic model

Variable	Value	95% CI or SE	Source
Male, %	59		DiRECT [8]
Age, years	54		DiRECT [8]
Remission (year 1) intervention, %	45.6	37.6, 53.0	DiRECT [8, 9]
Remission (year 2) intervention, %	35.6	28.2, 43.0	DiRECT [8, 9]
Remission (year 1) control, %	4.0	1.3, 7.4	DiRECT [8, 9]
Remission (year 2) control, %	3.4	0.7, 6.7	DiRECT [8, 9]
Relapse (year 2), %	28.4	18.7, 38.6	DiRECT [8, 9]
Mortality	NR^a^	NR^a^	Walker et al [18]
Cost of intervention (year 1), £	1137	1071, 1205	DiRECT [8, 9, 12, 17]
Cost of intervention (year 2, in remission), £	356	302, 413	Current study
Other costs intervention arm (year 1), £	689	537, 860	DiRECT [8, 9, 12, 17]
Other costs intervention arm (year 2), £	936	691, 1209	Current study
Other costs control arm (year 1), £	838	671, 1009	DiRECT [8, 9, 12, 17]
Other costs control arm (year 2), £	1582	1135, 2073	Current study
Annual cost of diabetes: year 1, £	1250	270	Roberts et al [21]
Annual cost of diabetes: increase per annum, %	6.7		Roberts et al [21]
HSU multiplier for diabetes	0.925	0.87, 0.96	Sullivan et al [20]

Distributions for probabilistic analysis: cost of diabetes, γ; HSU multiplier, β; mortality, β; other, bootstrap

HSU, health state utility

^a^Values are not reported due to the extent of data. See Walker et al [18] for the relevant data

## Results

### Within-trial results

**Fig. 1:**
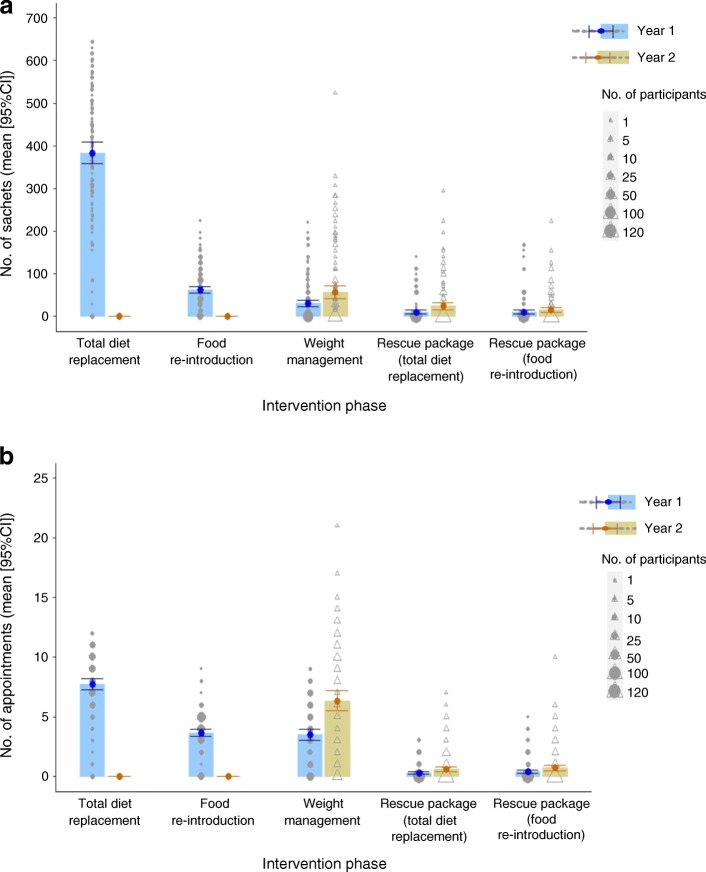
Mean use of low-energy-diet sachets (**a**) and health practitioner appointments (**b**) per participant, according to intervention phase. Error bars show 95% CI

During the first year of the DiRECT intervention, a mean of approximately 500 sachets of low-energy formula diet were issued to each participant, over 80% of the total 2 years’ consumption being consumed during the initial total diet replacement phase (Fig. 1). Each participant received a mean of 23 practitioner visits, the majority of these also during year 1. The cost of formula diet and practice visits together was £1364 (95% CI £1260, £1464) per participant entered over 2 years, and total intervention costs, including amortised clinic set-up costs, amounted to £1411 (95% CI £1308, £1511).

The focus and resource need of the Counterweight-Plus programme shifts from weight loss using total diet replacement in the initial 12–20 weeks to long-term weight maintenance, with data currently available for 2 years (Table 1, Fig. 1). Use of formula diet sachets in year 2 was limited to regular maintenance, for those who elected to use them, and for relapse management purposes (when participants regained over 2 kg body weight). Their total use amounted to less than 20% of the year 1 consumption, when they were used for total diet replacement, and decreased over the food reintroduction phase. Similarly, planned practitioner appointments in year 2, for maintenance and relapse management, were approximately 50% fewer than in year 1.

Participants in the intervention group had substantially fewer days on oral glucose-lowering and antihypertensive drugs. Over the 2 years, participants in the intervention group had a mean of 290 days on oral glucose-lowering medications, compared with 910 for those in the control group (difference 620 days; 95% CI 501, 738), and a mean of 381 days on antihypertensive medications, compared with 782 for those in the control group (difference 410 days; 95% CI 258, 537) (Table 3). The intervention group had fewer GP visits related to diabetes (difference 0.6 visits; 95% CI 0.2, 1.0). Other items of resource use were mostly lower in the intervention arm, including fewer hospital days. Lower use of glucose-lowering and antihypertensive medications, combined with fewer healthcare contacts, provided total savings in the intervention arm over 2 years of £796 (95% CI £150, £1465). This cost saving in routine (non-trial) resource use offset 56% of the 2 year intervention costs, leaving a 2 year incremental cost for the intervention of £616 (95% CI –£45, £1269) per participant entered. Given remission rates of 35.6% and 3.4% in the intervention and control arms, respectively, at 2 years, the resulting incremental cost per remission at 2 years was £1907 (lower 95% CI: intervention dominates, i.e. is cost-saving and more effective; upper 95% CI: £4212). Medication cost savings were principally due to lower use of glucose-lowering medicines (approximately 90% of total medication savings) and were divided equally between years 1 and 2. The difference in the total other non-intervention resource use (i.e. healthcare contacts and hospitalisations) in year 1 was negligible [17] and over the 2 years did not reach statistical significance; however, these cost savings were significant in year 2 (£521; 95% CI £12, £1085).

**Table 3:** NHS resource use and costs over the first 2 years of the DiRECT trial

Resource use items	Mean resource (SD)		Mean difference (95% CI)	Mean cost, £ (SD)		Mean difference, £ (95% CI)
	Intervention (*n* = 149)	Control (*n* = 149)		Intervention (*n* = 149)	Control (*n* = 149)	
Counterweight-Plus intervention (a)				1411 (593)	0	1411 (1308, 1511)
Medications (sum of individual drug days)^a^						
Oral glucose-lowering drugs (days)	290 (438)	910 (633)	−620 (−738, −501)	90 (221)	321 (486)	−231 (−314, −148)
Antihypertensive drugs (days)	381 (516)	782 (763)	−410 (−537, −258)	15 (29)	41 (94)	−26 (−42, −11)
Total cost of medications (b)				105 (222)	362 (486)	−257 (−337, −173)
Other resource use						
Primary and community care visits related to diabetes						
GP	1.1 (1.6)	1.7 (2.0)	−0.6 (−1.0, −0.2)	39 (58)	62 (75)	−24 (−38, −9)
Practice nurse	3.5 (3.2)	4.0 (2.6)	−0.6 (−1.2, 0.2)	37 (34)	43 (28)	−6 (−13, 2)
Healthcare assistant	0.8 (1.4)	0.9 (1.3)	−0.05 (−0.3, 0.3)	3.3 (5.6)	3.4 (5.1)	−0.2 (−1.4, 1.1)
Community care	0.8 (1.1)	0.7 (1.4)	0.09 (−0.2, 0.4)	32 (47)	34 (74)	−2.2 (−17, 11)
Primary and community care visits not related to diabetes						
GP	7.8 (8.5)	8.2 (9.6)	−0.4 (−2.3, 1.6)	285 (311)	301 (352)	−16 (−86, 59)
Practice nurse	1.7 (2.5)	2.4 (3.5)	−0.8 (−1.5, −0.1)	18 (27)	26 (37)	−8.6 (−16, −1.2)
Healthcare assistant	0.3 (0.9)	0.6 (1.9)	−0.3 (−0.6, −0.01)	1.3 (3.5)	2.5 (7.5)	−1.2 (−2.5, −0.1)
Community care	0.6 (1.4)	0.5 (2.3)	0.09 (−0.3, 0.5)	31 (71)	26 (120)	5.7 (−17, 27)
Outpatient visits	3.2 (4.1)	4.0 (5.1)	−0.8 (−1.8, 0.2)	500 (740)	594 (823)	−94 (−271, 86)
Hospitalisation^b^	0.7 (2.5)	2.1 (9.4)	−1.4 (−3.1, 0.01)	573 (1699)	966 (3109)	−392 (−964, 119)
Total cost of other resource use (c)				1519 (2242)	2058 (3665)	−539 (−1231, 84)
Total cost of resource use (b+c)				1624 (2268)	2420 (3690)	−796 (−1465, −150)
Total 2 year cost (a+b+c)				3036 (2346)	2420 (3690)	616 (−45, 1269)

^a^Aggregate drug days over all medications

^b^Cost of hospitalisation was estimated by matching reason for admission and recorded length of stay in DiRECT to the appropriate NHS reference cost (excluding excess bed-days where length of stay recorded in DiRECT exceeded the national average)

### Long-term cost-effectiveness

Across both arms of the trial, 79 participants achieved remission during the 2 year period, including five who did so during year 2. Of 74 participants in remission at year 1, 21 had relapsed by the end of year 2. At this observed annual relapse rate in DiRECT (28%), mean time to relapse would be approximately 3.5 years, with 13% of participants expected to remain in remission beyond 5 years. The resulting mean gain in life expectancy for a person in remission at 1 year is 0.30, and, for all participants entered in DiRECT, 0.13. Discounted QALYs were modelled to be increased by 0.06. Total costs relating to diabetes (other than ongoing intervention costs) were modelled, based on current treatment costs, ranging from approximately £15,000 for people remaining in remission until year 10 to £30,000 for those not in remission after 1 year. After accounting in the model for intervention costs and time to relapse, the intervention generated a £1337 cost saving (95% CI £674, £2081) per participant entered into the programme (Table 4). The intervention therefore dominated standard care and had the probability of being both cost-saving and cost-effective at a threshold of £20,000 per QALY of 1.00.

**Table 4:** Cost-effectiveness results: 2 year and lifetime analyses

Analysis	Intervention	Control	Incremental	95% CI
2 years				
Proportion of remission, %	35.6	3.4	32.3	23.5, 40.3
Costs, £	3036	2420	616	−45, 1269
Cost per 2 year remission, £			1907	Dominates, 4212
Lifetime				
Life-years	24.03	23.89	0.13	0.09, 0.20
QALYs	11.27	11.22	0.06	0.04, 0.09
Costs, £				
Intervention	1628^a^		1628	1439, 1835
Disease	31,319	34,283	−2964	−3706, −2308
Total	32,947	34,283	−1337	−2081, −674
Cost per QALY	Cost saving: intervention dominates			

^a^Total lifetime intervention cost including costs modelled for remission beyond year 2

**Fig. 2:**
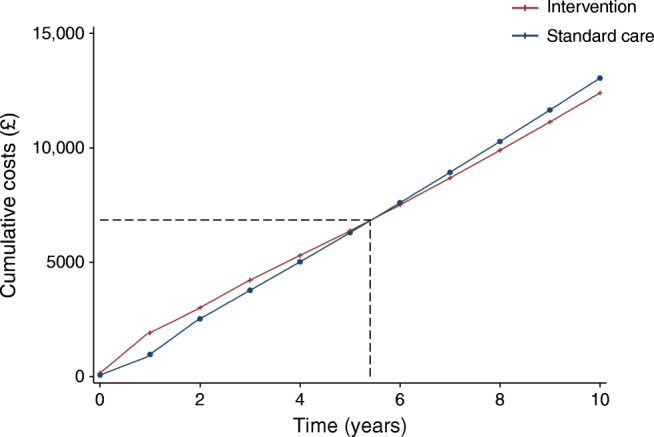
Cumulative predicted cost (per participant entered) for DiRECT/Counterweight-Plus intervention and standard care, up to 10 years. Dashed lines indicate financial break-even point

Follow-up of participants in DiRECT is continuing. As this analysis relies on 2 year data we estimated time to total cost equivalence (or break-even) and explored sensitivity to shorter times to relapse. The model predicted the intervention would become cost-saving overall after a period of 5–6 years under base case assumptions for relapse (Fig. 2). Assuming all people who achieved remission relapsed after a maximum of 3 years, the intervention remained cost-saving due to deferred diabetes (Table 5). Increasing the rate of relapse without modifying the maximum period of remission had a lesser impact on cost savings. The intervention also remained cost-saving when a reduced rate of remission after 1 year was assumed, and with a shorter time horizon for analysis. In these cases, although QALY gains were also reduced, the intervention remained dominant.

**Table 5:** Cost-effectiveness under alternative scenarios

Base case assumptions	Scenario	Incremental	
		QALY	Cost (£)
Base case result	–	0.059	−1337
All participants relapsed by 10 years	5 years	0.045	−839
	3 years	0.027	−165
Relapse rate as observed in trial	Accelerated relapse rate (×1.2)	0.053	−1117
Analysis time horizon – lifetime	10 years	0.027	−637
Remission rate as observed in trial	Year 1 remission rate × 0.8	0.047	−1001
Diminished QoL due to diabetes	No impact of diabetes	0.064	−1337
	Double impact of diabetes	0.054	−1337
Mixed population	Men only	0.080	−1864
	Women only	0.036	−847
SCI-Diabetes mortality (with diabetes)	Plus 10%	0.071	−1285
	Less 10%	0.047	−1391
Long-term diabetes costs	Plus 50%	0.059	−2421
	Less 50%	0.059	−252

Relapses were more frequent among women than among men in year 2. When the analysis was performed using sex-specific relapse rates, the overall QALY gain and cost were little different, although health gains and cost savings were more concentrated in men.

Assuming no detrimental impact of diabetes on QoL resulted in an increased QALY gain, the result of life expectancy being valued more highly. The model assumed that type 2 diabetes, with a diagnosis at about 55 years of age, and without intervention, would reduce life expectancy by 3.2 years in men and by 5.2 years in women. Reducing mortality rates for people with diabetes by 10% reduces the QALY gains from intervention to approximately 0.05. The intervention remained cost-saving both when excluding other healthcare cost savings in the early period and when substantially lower long-term costs due to diabetes were assumed.

## Discussion

It is most unusual to be able to provide a new medical treatment for a major chronic disease which is both health-improving and cost-saving. The DiRECT study has shown that durable remissions of type 2 diabetes to a non-diabetic state can be achieved through an integrated weight management programme, mostly for those achieving weight loss >10 kg, for almost half of all participants. Weight loss >15 kg in the intervention arm led to remissions for 86% at 1 year and 82% at 2 years [9]. The present analysis indicates that the intervention is likely to generate QALY gains and be not only cost-effective but also cost-saving after 5–6 years. Given the rate at which second-line glucose-lowering medication costs have been rising in recent years, cost-saving estimates may be conservative.

Although individuals with remissions are assumed to relapse to diabetes over time, cost savings were modelled to accrue beyond the point of relapse, with a delay in the requirement for more intense resource use due to diabetic complications. We have previously noted that roll-out of the intervention in routine practice may provide for efficiencies that could reduce costs; however, even under trial conditions, set-up costs are a minor component of total intervention costs, and no adjustment was made for such efficiencies in the present analysis.

There were fewer medical appointments for intercurrent medical problems in the DiRECT intervention group and fewer serious adverse events in year 2 [9]. This is in line with improved diabetes control and remissions for many. Better QoL would therefore be expected, and this was borne out by a general pattern of modestly improved EuroQol EQ-5D scores in the intervention group at both 12 and 24 months. [9] However, the mean duration of diabetes at baseline, of 3 years, is too short for the full impact of diabetes and its complications to have already impaired QoL. Therefore, QoL measurements over a 2 year period so early in the disease course cannot be expected to capture the long-term impact of more sustained diabetes remissions. Given that diabetes ultimately carries a major long-term burden for health-related QoL, we calculated QALY losses using a health state utility decrement due to diabetes based on the US Medical Expenditure Panel Survey [20]. Long-term health gains in our analysis are, however, attributable principally to improved life expectancy due to periods of remission, as our model assumed, perhaps conservatively, that over time all individuals in remission would return to the diabetes state by 10 years.

In the analysis of the Scottish Care Information Diabetes Database (SCI-Diabetes), life expectancy was found to be significantly lower in people with type 2 diabetes, irrespective of age group or socioeconomic status (with the exception of men >80 years in the most deprived quintile) [18]. These published Scottish survival rate data, on which estimates of life expectancy losses were based, are somewhat more conservative than the estimates from a large European study [22], which indicated that the years of life lost for people without known vascular disease would be about 5.2 years for men and 6.1 years for women.

An important assumption in our analysis was that remission returned participants to a life expectancy similar to that of the diabetes-free population. However, the benefit in terms of reduced mortality risk was modelled to be temporary, as all individuals in remission were assumed to relapse to diabetes within 10 years, and many were assumed to do so within 5 years. As yet, there are no published data from any country on the future health or life expectancy of people who achieve dietary--weight-loss-induced remissions of type 2 diabetes. Many of the participants in remission from diabetes in DiRECT had HbA_1c_ in the range of ‘prediabetes’ (42–48 mmol/mol [6.0–6.5%]). About 30–40% of the adult population have HbA_1c_ in this range, which is associated with progression to type 2 diabetes for perhaps a fifth, and poorer health outcomes than with lower HbA_1c_ [23–25]. The health outcomes for people in this range, which may be considered ‘post-diabetes’, after improving diet and lifestyle, are still unknown: they may be worse or better than those of the general population.

No subgroup effects have been proposed clinically, and we did not seek to do so. Relapse to diabetes in year 2, however, was significantly more likely among women than among men (*p*= 0.016). The SCI-Diabetes analysis found that women with diabetes lost more years of life compared with men [18]. A very large European study found a similar difference between sexes [22]. When we performed separate analyses for men and women, however, we modelled greater life expectancy gains in men than in women because of the lower rates of relapse in men, though life-year and QALY gains remained statistically significant for both; the intervention was cost-saving in both men and women, and the noted difference in relapse could be due to chance. These results should not imply withholding treatment for women, as they still did very well, but there may be benefit from modifying the intervention in the future to better support maintenance of weight loss and diabetes remission in women.

The present analysis is based on UK data both in terms of the DiRECT trial itself, and other data for costs of diabetes care under the NHS, and observed long-term mortality. Intervention costs in other countries may differ; though, as noted above, more efficient delivery, both in the UK and elsewhere, might be expected once the programme is established in routine practice. The major element of intervention cost is the formula diet, whose acquisition cost might fall in the future through economies of scale. Costs of routine diabetes care under clinical guidelines may be expected to increase with wider and earlier use of newer medications and an increasing duration of disease after younger onset.

A 2019 position statement issued by the joint Association of British Clinical Diabetologists and the Primary Care Diabetes Society [5] reviewed the current evidence for remission of type 2 diabetes, ranging from bariatric surgery, in 1987 [26], to the most recent evidence of dietary and behavioural interventions including DiRECT [9, 27, 28]. It concluded there was ample evidence to support the recommendation of achieving remission through weight loss, but that long-term follow-up was needed given the risk of weight regain. Relapse into diabetes, driven by weight regain, incurs costs from relapse management and from resumption of progressive costs for diabetes and its complications. Though relapse had a bearing on outcomes in our study, even relatively rapid relapse did not alter the conclusion that the low-energy diet intervention was capable of producing long-term health gains without adding long-term costs. The Counterweight-Plus intervention may therefore be expected to be transferable to other diabetes care settings in a similarly cost-effective manner.

## Data Availability

Once the data collection and planned analyses of DiRECT are complete (expected 2024), anonymised participant level data will be shared on reasonable request. The study protocol and statistical analysis plan may be obtained from the corresponding author.

## Abbreviations

DiRECT: Diabetes Remission Clinical Trial
GP: General practitioner
NHS: National Health Service
QALY: Quality-adjusted life-year
QoL: Quality of life
SCI-Diabetes: Scottish Care Information Diabetes Database
